# External Glandular Enlargements

**Published:** 1877-12

**Authors:** Z. Collins McElroy

**Affiliations:** Fellow Zanesville Academy of Medicine; Member Muskingum County Medical Society, etc., etc.


ART. II.—*External glandular enlargements. The physiology of the Lymph system of human bodies.* By Z. COLLINS MCELROY, M. D. Fellow Zanesville Academy of Medicine; Member Muskingum County Medical Society, etc., etc.

Read before the Muskingum County Medical Society, at Zanesville, O., October, 1877.

When I proposed to the Society to take up the subject of "External glandular enlargement," I had in my mind the enlargement of the cervical glands of young children, a by no means uncommon occurrence in my observation. And most likely so to others. I purposely avoided the use of the term by which they are commonly designated, as one calculated to give offense in private practice and believing that nothing will be lost by dropping it out of use. For, it is difficult to think one way, and speak another without being occasionally caught in the somewhat disagreeable predicament in which it places any one who attempts such a duplex performance.

But I am satisfied my conception at that moment was too narrow —for, it should have been "enlargement of Lymph glands," with the addition of the larger salivary glands about the face and neck. Using the term gland in its wide sense, it would embrace liver, lungs, kidneys, etc., of the more massive structures, which is by no means the intent, or purpose of the Society now. Let it be understood, then, that our discussion will embrace the enlargement of the external Lymph and Salivary glands—without reference to location, whether about face and neck, in the axilla, or groin.

I am one of those who think that some knowledge of the anatomy, as well as the physiology of any gland, or structure, is essential to a proper understanding of its pathological or modified status. In speaking exactly, pathology is, modified physiology.

It is well, also, to understand that perfect physiological conditions of living tissue, glandular, or otherwise, are necessarily, and to some extent, at least, an ideal standard, examples of which are not very common in living beings. I long since learned that old things, partly worn out, or damaged, lasted much longer than new and perfect things. I gave, several years since to one of my poor neighbors, a suit of clothes which I had worn a single summer, which were, for my purposes, considered worn out. He has had them three or four years in nearly constant use, and they are not wholly useless yet, though in a much greater state of dilapidation than when I parted with them.

So it really is in organic life. A structure more or less modified from an ideal physiological standard, may be expected, and actually does acceptable duty for a much longer period than one ideally perfect. I purpose now to confine myself mainly to the physiology,—the use of, and what the Lymph apparatus actually does in living bodies.

What is known of the gross anatomy of the Lymphatic system is accessible in text books, and would be out of place here. For the minute, or microscopical nature and character of Lymph tissue, special treatises must be consulted ; they are very modern; almost of to-day, and not very interesting to the actual working practitioner—therefore, not found in ordinary medical libraries. But one feature disclosed by microscopists is not without interest, and that is the identity of structure, histologically of serous membranes and Lymph glands. Serous membranes are lymph glands spread out. This fact in connection with the serious nature of active pathological conditions of serous membranes—known as inflammatory, sheds some reflected light on the general physiology of the structures, to which attention will be called at a proper time.

Ordinary methods of physiological study of structures have absolutely failed to throw any light on the uses of this lymphatic system up to this time, when compared with its extent and complexity in the human body. In fact, it is about the only structure histologically considered, the mystery of whose function, remains intact to-day. I speak in this sense of the profession, and learning of the world in the aggregate, not individuals.

A function has been definitely assigned to a portion of the lymphatic system,—that in the abdomen—a different name given, and the thing considered settled, but it won't stay settled. Modern microscopical researches have rudely thrust such settlements aside. And any other settlement which does not embody the facts of nature, will ultimately share the same destiny.

Some years ago the question "How are the structures of a living body reproduced from new material, preserving personal identity through life?" came up for settlement in the course of my investigations of the phenomena of life from a physical stand point.

Elsewhere in nature every special thing of life, during life or just before, or, in the act of death, so to speak, makes provision for its own perpetuation and multiplication. Animal life makes provision for its own perpetuation, in gross during life. With fishes and fowls, by eggs; with the higher forms of life, by carrying embryos in the bodies of females up to a certain point of development—then they are born, and have the care of the parents a longer or shorter time, before entering upon individual independent life.

Examine an egg, a chicken's egg, with which we are, perhaps, most familiar. What do you find? A hard calcareous skin, or external support. Inside of the calcareous envelope a strong membraneous envelope. Here, a clear, ropy albuminous fluid, another delicate envelope with a much denser enclosure of varying yellowish shades of color.

Artificial incubation has shown that nothing is lacking for the production of a perfect young chick, except a steady and elevated range of temperature. Inside of the calcareous envelope of the egg there is the material which, with the assistance of the steady range of external heat, arranges itself in the form of a living chick, occupying, about 21 days. Expressed in a formula—the egg must be held to be; 1st, so much material as needful for: 2d, storing up the force: 3d, and to form the structures of the new chick, up to that point in life at which it can appropriate other, and new material, to its future growth and evolution.

In the vegetable world, each special form of vegetable kind, stores up the force for its own reproduction, multiplication and perpetuation, in a seed, or tube, exactly analagous in its purposes and results, to the egg of the chick.

These facts, it seems to me, throw a good deal of light on the question "How are the structures of a living body reproduced from new material during life, so as to preserve personal identity?"

The conclusion is irresistible, that by some analagous process, each special structure be it viscus or tissue, in the act of functional decay, "stores up" the force, in the necessary material, to carry its own, and new material, with appropriate and necessary conditions up to the forms of structure from which it was derived.

At all events such a conclusion is in harmony with other, and like processes in other forms of life, and with very much greater probability of its being true, than a reference of it to the operations of an unknown and hypothetical vital force.

The Lymph system, then, becomes by this conception, "the fountain of life." For the lymph is the eggs' seed, or germs, of the several structures of the body from which it is taken, or in which it is found, or derived.

A closer scrutiny will disclose that the lymph system is, in each living body, a unity, serving the same purposes and ends whenever found. That to fulfill the office, or duties here assigned to it, it must have the exact anatomy it has in the human body. That the point at which it is added to the blood stream is the exact and only point at which it should be poured into it, viz: just before entering the heart on its path to the lungs. Here it is mingled with the old, and new material introduced through the stomach, meeting in the lungs the inhaled atmosphere where the bulk of the changes are effected, which fits the material to ascend in chemical complexity, to form the structures of living bodies.

True, this view will necessitate considerable modification of currently accepted physiology of so called "digestion," if that means the bulk of the chemical changes which fits new material to become living flesh. And such modification will greatly simplify our conceptions of "digestion."

The Lymph system with all its complexity and extent, will have a function, according to such a view, corresponding with its anatomical extent, and complexity of structure, viz: to collect from the debris of the tissues, the material in which the several structures have stored up the force for their own reproduction from new material during the life of each individual. The old is by this means, constantly assisted with the new material steadily going into living bodies, so that personal identity, the indisputable posession of each individual, becomes intelligible.

The Lymph is the eggs, seeds, or germs, of the living structures from which it is derived.

The structures of every living body are constantly, during life, undergoing the process of disintegration, waste or decay. As constant a stream of new material, organic, and inorganic, is as steadily going in from the exterior. To repair the structures as they are constantly and momentarily wasted, the supply of seed must be continuous, to insure continuous reproduction. This is the exact condition the Lymph fulfills; varying only as it is needed, in quantity, or as supplied by waste.

It is seen, then, that the Lymph glands perform a most important function in animal organic life. Do the pathological states of the glands correspond with this important conception of their uses? most assuredly they do, for, from the remotest antiquity, pathological changes have been regarded as of the most serious nature.

The most obvious changes are enlargements and suppurations, not alone of the larger or smaller glands, but of the tissues generally, especially the bony, ligamentous, and cartilaginous. Here changes have been known, popularly, from the long ages of the past, as "the King's evil," an omnious, yet, in the light of modern investigations, truthful conception of the reality behind them. And if the source of life—the Lymphatic system—be associated in our minds with the Kingly, or Regal function which it is here shown to possess, could any popular name be more appropriate, or more significant, than the "King's Evil." Affections of the Lymph glands are now regarded as constitutional, an organic defect, seldom or never wholly remediable. A condition of cachexia—depravity of the gravest, and most serious character.

The pathology and remedial management of pathological states of the Lymph apparatus may well engage our attention, for, they lie at the very foundation of our own lives, and the lives of our fellow beings.

I had purposed confining myself to the Physiology of the glandular structures under consideration, yet I trust you will bear with me while I say something in regard to symptomatology of glandular affections.

Very little is known in regard to pathological symptoms of the

Lymph glandular structures, until by their increase in size they attract attention. There seems to be no special sensibility pertaining to them, and not any considerable degree of common sensibility—less certainly than some other structures. These glands, like the invisible forces of nature, do their work in secrecy and silence and show nothing except results. Certainly, enlargements of glandular structures are not what we could call painful, except by mechanical displacement, or pressure. There is, it is true, always more or less disturbance of the general health. Young children are certainly more peevish and fretful; sometimes with temperature above natural, but more frequently just natural. Perhaps I might say as the results of very recent observations, that in the early stages of enlargement there is an elevation, but, under treatment soon settles down to the natural. I have had very recently three cases under observation and professional management, all improving, but not yet well—that is the enlargements of the glands have not yet subsided.

I may, also, be permitted to say, that, as the pathology of the Lymph apparatus is more attentively studied, I think a good deal of light will be thrown upon the phenomena of tuberculosis, cancer, tumors, etc., as well as on certain varieties of chronic skin eruptions, especially on leprosy, elephantiasis, etc.

